# A Treatise on Rheumatic Gout

**Published:** 1858-09

**Authors:** 


Art. IX.—*A Treatise on Rheumatic Gout, or Chronic Rheumatic Arthritis of all the Joints.* By Robert Adams, M.D., Surgeon to the Richmond Hospital, etc. Illustrated by wood-cuts and an Atlas of plates. 8vo., pp. 362. London: Churchill, 1857.

During the past few years there seems to have been a chronic *cacoethes scribendi* among medical men, more especially abroad. Without throwing any new light on the morbid anatomy and treatment of disease, the object of some authors appears to have been merely to show the public how old facts could be dressed up and reproduced under a new garb. Of the numerous works, however, which we have seen on the subject of gout and rheumatism, there has been none to which we more cordially extend a warm welcome than to the one before us. Dr. Adams has made good use of his opportunities, and has acquired a high reputation by his many valuable contributions to surgical and pathological science. The volume now before us consists of fourteen chapters; several of which are devoted to the history, pathology, symptoms, and treatment of this affection; but by far the greater number are taken up with the discussion of the disease as it occurs in the different articulations. Some of these descriptions would in themselves form valuable essays, and do credit to any author. The style is clear and accurate, and the work is illustrated by excellent drawings and interesting cases; it is, in fact, one of which he and his countrymen may justly be proud. We heartily commend it to the perusal of our readers, and trust we shall be able, at no distant day, to present them with a critical analysis of its contents.

